# Falls and associated risk factors in a sample of old age population in Egyptian community

**DOI:** 10.3389/fpubh.2023.1068314

**Published:** 2023-01-26

**Authors:** Abd El Hamied Ibrahim El Sayed, Mohamed T. Said, Omnia Mohsen, Aziza M. Abozied, Mohamed Salama

**Affiliations:** ^1^Department of Occupational Therapy, National Institute of Longevity Elderly Sciences NILES, Beni-Suef University, Beni Suef, Egypt; ^2^Physical Therapy for Elderly, National Institute for Longevity Elderly Sciences, Beni-Suef University, Beni Suef, Egypt; ^3^Medical Anthropology, National Institute of Longevity Elderly Sciences NILES, Beni-Suef University, Beni Suef, Egypt; ^4^Community Health Nursing, Beni-Suef University, Beni Suef, Egypt; ^5^Institute of Global Health and Human Ecology (IGHHE), The American University in Cairo, Cairo, Egypt; ^6^Faculty of Medicine, Mansoura University, Dakahleya, Egypt; ^7^Atlantic Senior Fellow for Equity in Brain Health at the Global Brain Health Institute (GBHI), Trinity College Dublin (TCD), Dublin, Ireland

**Keywords:** risk factors, old age, Egyptian aging study, falls, AL-SEHA

## Abstract

**Introduction:**

Falling is a major health problem among old age persons and are the sixth cause of mortality and morbidity among them. Assessing the prevalence of falls among elderly in an Egyptian community and investigating its associated risk factors using the Arabic translation of the SHARE-Questionnaire.

**Subjects and methods:**

This cross-sectional analytic study was a part of the pilot for AL-SEHA project. It included 289 old age people (50+ years age) residing in the study areas. The main project data were collected using the Arabic translation of the SHARE (Survey of Health, Aging, and Retirement in Europe) questionnaire. The original project data were collected by investigators from five universities, then uploaded to the internet server domain of the American University in Cairo (AUC) Social Research Center.

**Results:**

The prevalence of falls was 11.07% (95% CI: 7.95–15.21). Falls were significantly more among 70 years or older (*p* < 0.001), unemployed or housewives (*p* = 0.026), have a family caregiver (*p* = 0.022), and home facilities for disability (*p* = 0.015). They had significantly higher rates of ischemic heart disease, hypertension, dyslipidemia, stroke, and diabetes mellitus. The most frequently reported problems were the fear of fall and dizziness (62.5%). The multivariate analysis identified the history of stroke and diabetes mellitus, the fear of fall and dizziness, and the total number of health problems and the score of difficulty in performing physical activities as significant independent predictors of fall occurrence. The history of stroke was the strongest risk factor (OR 33.49, CI: 3.45–325.40).

**Discussion and recommendations:**

The prevalence of falls among old age persons in the studied community is not alarmingly high. It is highest among stroke patients. Community interventions and rehabilitation programs are recommended to train and educate old age people, especially those at risk such as stroke and diabetic patients, and those with dizziness to improve their physical fitness and reduce the fear of fall among them.

## Introduction

Falling is a common incident and a major health problem among old age persons. The World Health Organization (WHO) reports indicate that worldwide more than half-million falls with fatality occur yearly, mostly among persons 65 years age or older (1). Falls and consequent injuries and associated complications are the sixth cause of mortality and morbidity among elderly. They account for more than one-third of all deaths due to injuries and are a leading cause of related mortality among those 65 years or older in the United States. Moreover, approximately one-third of the old age persons 65 years or older and living in community dwellings experience one fall incident per year (2). The problem has deleterious consequences on fallers, their families, and the community at large (3).

Research addressing falls in old age attributes its high incidence to a combination of several intrinsic as well as extrinsic factors (4). Among the intrinsic risk factors identified are those related to old age persons' health status often associated with chronic diseases and polypharmacy, in addition to visual, auditory, and equilibrium problems, as well as the psychological disorders as the symptoms of depression. The extrinsic factors are mostly related to environmental hazards (5, 6).

Several environmental factors have been identified as risk factors underlying falls in the old age population (7). Among these are the slippery floors whether ceramic or old rugs, tripping obstacles, and poor lighting. Other factors include disordered furniture, ill-designed footsteps, broken stairs, and lack of handrails (8). Added to these are the lack of safety measures like grab rails, as well as first-aid and other indoor and outdoor environmental factors (9).

The fear of falling (FOF) has also been identified as an intrinsic factor of considerable importance in the incidence of falling among the elderly. Thus, it has been demonstrated that the prevalence of FOF is significantly higher among those old age persons who experienced falling in comparison with those with no history of falls. Moreover, those who fear falling tend to limit their physical activities and this in turn leads to reduction in their quality of life (10). Furthermore, research showed that the old persons 65 years or older with history of falls have a higher probability of falling again (2).

Falling and consequent injuries and complications among old age people constitute a major burden for them, their caregivers, the society, as well as the system of healthcare. The problem gains more importance with the demographic changes in Egypt with increasing numbers and proportion of elderly in the population. Thus, measuring the magnitude of the problem and identifying its underlying risk factors is of importance in mitigating such burden.

This study is a part of the pilot of the Egyptian aging study (AL-SEHA) which is the first of its type in Egypt. The current study aimed at assessing the prevalence of falls among elderly in an Egyptian community and investigating its associated risk factors using the SHARE-Questionnaire. The SHARE survey and its updated questionnaires can be accessed from this link: http://www.share-project.org/home0.html. The research questions are: (1) What is the prevalence of falls among Egyptian old age persons? and (2) What are the significant risk factors associated with these falls? The outcomes of this work should be advising policies to decrease the rate of falling among seniors through understanding of the current situation and risk factors in Egypt.

## Subjects and methods

### Research design

A cross-sectional analytic study was used where all dependent and independent variables are assessed at one and the same point in time.

### Setting

The study is a part of a DAAD funded project (Aging in the East Mediteranean Region: Emage) which is piloting the Egyptian Longitudinal Aging Study (AL-SEHA).

### Participants

Old age people (50+ years age) residing in the study areas constituted the sampling population for the study. The inclusion criteria were being able to communicate, and living independently at home, in rented accommodation, in a hostel, or in a retirement home. Those having a gross psychiatric disturbance were excluded. The sample consisted of 289 old age persons. This sample size was large enough to estimate an expected fall prevalence rate of 11% or higher with 4% absolute precision at 95% level of confidence and 80% power. A non-probability convenience sampling technique was used in recruiting participants according to the eligibility criteria.

### Data collection tool

The main project data were collected using the Arabic translated version of the SHARE (Survey of Health, Aging, and Retirement in Europe) questionnaire. It was validated by expertise from five Egyptian universities and the Social Research Center (SRC) American university Egypt. This is an extensive interview questionnaire developed by Mehrbrodt et al. (11). It consists of 16 sections covering respondent's personal and family information, health status and physical activity, mental abilities, mental health, health care, work and retirement, physical testing, relations with children, social support, finances, living conditions, family income, family assets, social activities, in addition to two sections for the data collector's views and information.

### Fieldwork

The original project data were collected by investigators from five universities who were trained in interviewing using the prepared SHARE form translated into Arabic. The sample of eligible old age persons was selected. The investigators in each governorate contacted the potential participants in their area, explained to them the aim of the study and the procedure of data collection. They were invited to participate after being informed about their rights. Those who gave their consent to participate were asked for the suitable time to conduct the interview. At the set time, the investigators visited participants at their homes. They were interviewed using the data collection form. The procedure of data collection lasted for about 1 month. Each investigator was assigned 15 old age person for interviewing. The collected data were then uploaded to the internet server domain of the American University in Cairo (AUC) Social Research Center.

In the present study, data pertaining to the old age persons residing in Beni Suef were extracted from the main project dataset. These data included parts of the personal and family information section such as age, gender, residence, education, etc.; parts of the health status and physical activity section as the history of chronic diseases, health problems, medication administration, vision, and hearing, etc.; mental abilities as recall; mental health as sleep problems; health care utilization; job; physical testing as grip; relations with children as caregiving; as well as the living conditions and facilities.

### Statistical analysis

The data were extracted from the SPSS (Version 25) dataset. Bivariate analyses included chi-squared or Fisher exact tests for the comparison of categorical risk factors between faller and non-faller groups. Numeric data were compared using independent *t*-tests or Mann-Whitney tests as suitable. Multiple logistic linear regression with backward Wald method and ROC (Receiver Operator Curve) analysis were used to identify the independent risk factors of falling and model its risk. Statistical significance was considered at *p* < 0.05.

## Results

In a sample of 289 seniors, 32 reported fall incidents indicating a prevalence rate of 11.07% (95% CI: 7.95–15.21). Table 1 indicates that the old age people who experienced falls were significantly more among those aged 70 years or older (*p* < 0.001), unemployed or housewives (*p* = 0.026), have a family caregiver (*p* = 0.022), and home facilities for disability (*p* = 0.015). Their percentages also tended to be higher among females although the difference was not statistically significant (*p* = 0.170).

**Table 1:** Comparison of the demographics of old age persons with and without fall history.

	**Fall group**				**Chi-squared test**	***p*-value**
	**No (*****n*** = **257)**		**Yes (*****n*** = **32)**			
	**No**.	**%**	**No**.	**%**		
**Age**						
< 60	136	52.9	9	28.1		
60–	78	30.4	9	28.1		
70+	43	16.7	14	43.8	14.066	< 0/001^*^
Mean ± SD	60.6 ± 8.0	67.6 ± 10.6		
**Gender**						
Male	113	44.0	10	31.3		
Female	144	56.0	22	68.8	1.883	0.170
**Residence**						
Urban	157	61.1	20	62.5		
Rural	100	38.9	12	37.5	0.024	0.877
**Education**						
None	36	14.0	8	25.0		
Basic/intermediate	98	38.1	13	40.6		
University	123	47.9	11	34.4	3.420	0.181
**Job**						
Working	111	43.2	6	18.8		
Retired	57	22.2	9	28.1		
Unemployed/housewife	89	34.6	17	53.1	7.293	0.026^*^
Family caregiver	29	11.3	9	28.1	Fisher	0.022^*^
Live alone	39	15.2	7	21.9	0.954	0.329
**Crowding index**						
< 2	240	93.4	31	96.9		
2+	17	6.6	1	3.1	Fisher	0.704
Mean ± SD	0.94 ± 0.55		0.82 ± 0.56		1.177	0.246
**Water closet**						
Private	256	99.6	32	100.0		
Shared	1	0.4	0	0.0	Fisher	1.000
Home facilities for disability	19	7.4	7	21.9	Fisher	0.015^*^
**Building**						
1–2 floors	196	76.3	25	78.1		
3+ floors	61	23.7	7	21.9	0.055	0.815
**Family financial ability**						
Very low	28	10.9	3	9.4		
Low	58	22.6	10	31.3		
Moderate	145	56.4	15	46.9		
High	26	10.1	4	12.5	1.597	0.660

^*^Statistically significant at *p* < 0.05.

As illustrated in Table 2, one-half of the old age persons in both groups were obese. Those in the fall group had significantly poorer visual acuity (*p* = 0.005) and hearing (*p* = 0.013), and less exercise frequency (*p* < 0.001). They also had lower recall rates for words (*p* = 0.028) with more depression (*p* = 0.002), use of antidepressants (*p* = 0.015), more sleep problems (*p* = 0.001), and hospital admission (*p* = 0.041). Their scores of difficulty in performing physical and daily life activities were higher (*p* < 0.001).

**Table 2:** Comparison of health status of old age persons with and without fall history.

	**Fall group**				**Chi-squared test**	***p*-value**
	**No (*****n*** = **257)**		**Yes (*****n*** = **32)**			
	**No**.	**%**	**No**.	**%**		
**BMI** ^#^						
Normal (18.5– < 25.0)	40	20.4	7	31.8		
Overweight (25.0– < 30.0)	58	29.6	4	18.2		
Obese (30.0+)	98	50.0	11	50.0	2.802	0.423
Mean ± SD	30.95 ± 6.9		30.08 ± 6.2		0.608	0.548
Use eyeglasses	94	36.6	16	50.0	2.175	0.140
**Visual acuity**						
Excellent	38	14.8	3	9.4		
Very good	86	33.5	5	15.6		
Good	91	35.4	10	31.3		
Poor	32	12.5	10	31.3		
Very poor	10	3.9	4	12.5	14.877	0.005^*^
Use hearing aid	7	2.7	0	0.0	Fisher	1.000
**Hearing**						
Excellent	61	23.7	6	18.8		
Very good	95	37.0	11	34.4		
Good	77	30.0	10	31.3		
Poor	22	8.6	2	6.3		
Very poor	2	0.8	3	9.4	12.712	0.013^*^
Ever smoker	50	19.5	10	31.3	2.406	0.121
Current smoker	26	10.1	5	15.6	Fisher	0.361
**Physical exertion frequency**						
>1/week	139	54.1	10	31.3		
1/week	60	23.3	3	9.4		
1–3/month	18	7.0	4	12.5		
Rare	40	15.6	15	46.9	21.215	< 0.001^*^
Recall of words (mean ± SD: max = 10)	4.33 ± 1.96		3.38 ± 2.27		2.280	0.028^*^
Felt depressed	111	43.2	23	71.9	9.415	0.002^*^
Used antidepressants	24	9.3	8	25.0	Fisher	0.015^*^
Have sleep problems	124	48.2	25	78.1	10.170	0.001^*^
Hospital admission	28	10.9	8	25.0	Fisher	0.041^*^
Have difficulty getting aids	4	1.6	0	0.0	Fisher	1.000
Good grip	235	91.4	29	90.6	Fisher	0.747
**Walking ability**						
High	221	86.0	23	71.9		
Moderate	10	3.9	4	12.5		
Low	26	10.1	5	15.6	5.830	0.054
**Score of difficulty with**						
Physical activities (max = 10)	3.11 ± 3.08		6.53 ± 2.26		7.718	< 0.001^*^
Daily life activities (max = 13)	1.38 ± 2.23		4.59 ± 3.91		4.557	< 0.001^*^

^*^Statistically significant at *p* < 0.05.

^#^Missing data.

Table 3 demonstrates that the old age persons in the fall group reported significantly higher rates of ischemic heart disease (*p* < 0.001), hypertension (*p* = 0.015), dyslipidemia (*p* = 0.042), stroke (*p* = 0.008), and diabetes mellitus (*p* < 0.001). They also had higher rates of osteoporosis (*p* = 0.031), cataract (*p* < 0.001), and arthritis (*p* < 0.001), in addition to more history of fracture hip (*p* = 0.016). Overall, the mean number of chronic diseases was higher among them (3.38 ± 1.77) compared with those in the no-fall group (1.74 ± 1.63), and the difference was statistically significant (*p* < 0.001).

**Table 3:** Comparison of the prevalence of chronic diseases reported by old age persons with and without fall history.

**Diseases**	**Fall group**				**Chi-squared test**	***p*-value**
	**No (*****n*** = **257)**		**Yes (*****n*** = **32)**			
	**No**.	**%**	**No**.	**%**		
Ischemic heart	35	13.6	13	40.6	14.985	< 0.001^*^
Hypertension	118	45.9	22	68.8	5.941	0.015^*^
Dyslipidemia	55	21.4	12	37.5	4.142	0.042^*^
Stroke	8	3.1	5	15.6	Fisher	0.008^*^
Diabetes mellitus	68	26.5	19	59.4	14.653	< 0.001^*^
Bronchial asthma	15	5.8	0	0.0	Fisher	0.388
Pulmonary disease	8	3.1	0	0.0	Fisher	0.604
Neoplasm	7	2.7	0	0.0	Fisher	1.000
Osteoporosis	33	12.8	9	28.1	Fisher	0.031^*^
Genitourinary	16	6.2	3	9.4	Fisher	0.452
Parkinsonism	2	0.8	0	0.0	Fisher	1.000
Cataract	21	8.2	11	34.4	Fisher	< 0.001^*^
Arthritis	62	24.1	14	43.8	5.655	0.017^*^
Total number (mean ± SD)	1.74 ± 1.63		3.38 ± 1.77		4.951	< 0.001^*^
History of fracture hip	6	2.3	4	12.5	Fisher	0.016^*^

^*^Statistically significant at *p* < 0.05.

Concerning the health problems reported by old age persons. Table 4 indicates statistically significantly higher percentages among those in the fall group in all types of problems. The only exception was that of recurrent cough (*p* = 0.080). The most frequently reported problems among them were the fear of fall and dizziness (62.5%), while the lowest were recurrent cough (15.6%) and incontinence of urine (21.9%). The table shows that all those in the fall group had at least one reported problem compared to 62.3% of those in the no-fall group (*p* < 0.001). Overall, the mean number of health problems was higher among those in the fall group (5.34 ± 1.81) compared with those in the no-fall group (1.39 ± 1.53), and the difference was statistically significant (*p* < 0.001).

**Table 4:** Comparison of health problems reported by old age persons with and without fall history.

**Health problems**	**Fall group**				**Chi-squared test**	***p*-value**
	**No (*****n*** = **257)**		**Yes (*****n*** = **32)**			
	**No**.	**%**	**No**.	**%**		
Back pain	128	49.8	26	81.3	11.304	0.001^*^
Angina	32	12.5	12	37.5	Fisher	0.001^*^
Dyspnea	34	13.2	11	34.4	Fisher	0.004^*^
Recurrent cough	17	6.6	5	15.6	Fisher	0.080
Edema lower limbs	38	14.8	17	53.1	27.145	< 0.001^*^
Sleep disorders	44	17.1	21	65.6	38.404	< 0.001^*^
Fear of fall	14	5.4	20	62.5	Fisher	< 0.001^*^
Dizziness	27	10.5	20	62.5	56.491	< 0.001^*^
Incontinence of urine	22	8.6	7	21.9	Fisher	0.028^*^
Have at least 1 problem	160	62.3	32	100.0	18.180	< 0.001^*^
Mean ± SD	1.39 ± 1.53		5.34± 1.81		−11.873	< 0.001^*^

^*^Statistically significant at *p* < 0.05.

Similarly, the old age persons in the fall group had higher percentages of administration of almost all types of medications, reaching statistical significance regarding medications for IHD and other cardiac diseases (*p* = 0.001), diabetes (*p* < 0.001), arthritis (*p* = 0.010), analgesia (*p* = 0.005), and bronchitis (*p* = 0.035) as presented in Table 5. The table also indicates that all those in the fall group had at least one medication administered compared to 78.2% of those in the no-fall group (*p* = 0.003). Overall, the mean number of medications was higher among those in the fall group (4.00 ± 2.09) compared with those in the no-fall group (2.16 ± 1.96), and the difference was statistically significant (*p* < 0.001).

**Table 5:** Comparison of the administration of medications as reported by old age persons with and without fall history.

**Medications for**	**Fall group**				**Chi-squared test**	***p*-value**
	**No (*****n*** = **257)**		**Yes (*****n*** = **32)**			
	**No**.	**%**	**No**.	**%**		
Dyslipidemia	48	18.7	9	28.1	1.604	0.205
Hypertension	117	45.5	20	62.5	3.289	0.070
IHD	28	10.9	11	34.4	Fisher	0.001^*^
Other cardiac	14	5.4	8	25.0	Fisher	0.001^*^
Bronchial asthma	13	5.1	0	0.0	Fisher	0.374
Diabetes mellitus	63	24.5	18	56.3	14.208	< 0.001^*^
Arthritis	65	25.3	15	46.9	6.622	0.010^*^
Analgesics	87	33.9	19	59.4	7.981	0.005^*^
Sleep pills	14	5.4	4	12.5	Fisher	0.124
Antidepressants	4	1.6	2	6.3	Fisher	0.134
Osteoporosis (hormonal)	25	9.7	6	18.8	Fisher	0.130
Osteoporosis (other)	6	2.3	1	3.1	Fisher	0.564
Antacids	44	17.1	7	21.9	0.443	0.506
Bronchitis	26	10.1	8	25.0	Fisher	0.035^*^
Take at least 1 medication	201	78.2	32	100.0	8.649	0.003^*^
Mean ± SD	2.16 ± 1.96		4.00 ± 2.09		−4.730	< 0.001^*^

^*^Statistically significant at *p* < 0.05.

The multivariate analysis (Table 6) demonstrates that the statistically significant independent predictors of fall occurrence are the history of stroke and diabetes mellitus, the fear of fall and dizziness, in addition to the total number of health problems and the score of difficulty in performing physical activities. It is evident that the history of stroke is the strongest risk factor for falling, increasing its risk more than 33 folds (OR 33.49, CI: 3.45–325.40).

**Table 6:** Best fitting multiple logistic regression model for the occurrence of fall.

	**Wald**	**Df**	***p*-value**	**OR**	**95.0% CI for OR**	
					**Upper**	**Lower**
Constant	31.334	1	0.000	0.00		
Stroke	9.160	1	0.002	33.49	3.45	325.40
DM	7.032	1	0.008	7.42	1.69	32.61
Fear of fall	6.656	1	0.010	7.14	1.60	31.78
Dizziness	4.653	1	0.031	5.08	1.16	22.23
No. of health problems	16.113	1	0.000	2.72	1.67	4.42
Score of difficulty in physical activities	5.124	1	0.024	1.33	1.04	1.71

Nagelkerke *R* Square: 0.74.

Hosmer and Lemeshow Test: *p* = 0.181.

Omnibus Tests of Model Coefficients: *p* < 0.001.

Variables entered on step 1: Age, gender, sleep problems, IHD, dyslipidemia, hypertension, stroke, diabetes, fracture hip, fear of fall, dizziness, urine incontinence, No. of problems, score of difficulty with physical activities and ADL, exercise frequency, recall of words, depression.

## Discussion

This study was aimed at assessing the prevalence of falls among elderly in an Egyptian community and investigating its associated risk factors using the SHARE-Questionnaire. The findings indicate a relatively high prevalence, with stroke being the most important risk factor underlying falls in this population.

According to the current study results, slightly more than one-tenth of the old age persons in the sample gave a positive history of previous fall incidents. This is quite a high rate that needs to be addressed carefully given its untoward consequences on the elderly person and his/her family, as well as the whole community and health care system. Nonetheless, the rate is still lower than that reported by Geetha et al. (12) in a study of fall risk in India who reported a rate 27.6%. This discrepancy might be due to that the Indian study was in a rural area, and the age of their participants was older in comparison with our study. Similarly, a study in China reported a prevalence rate of 20.65 among elderly persons living in rural and urban community dwellings (13). Their age group was also more than one decade older in comparison with the present study.

The bivariate analyses of the current study identified significant associations between certain old age persons' age, and being unemployed, retired, or housewife. Additionally, there was a trend of higher prevalence among women. In agreement with this, a study in New Zealand found significant associations with older age and female gender (14). On the same line, Guerreiro et al. (15) in a study in Spain demonstrated that female gender was a significant predictor of falls among old age people. However, the multivariate analysis could not confirm a significant independent role in the risk of falling.

Meanwhile, factors related to old age persons' health status and identified in bivariate analyses as having significant associations with the occurrence of falls were poor vision and hearing, lack of physical activity, sleep problems, inadequate recall, depression symptoms, and previous hospital admission. In addition, the difficulty in performing physical and daily life activities was a significant risk factor. These are all indicative of poorer health status that may lead to higher risk for accidents and injuries. They also reflect a low physical fitness leading to inability to prevent falling in case of stumbling or losing equilibrium. Similar associations between old age persons' falls and poor vision or hearing impairment (16, 17), sleep disorders (18), depression (19), and impaired recall and mental abilities (20) were previously reported.

Nevertheless, only the score of difficulty in performing physical activity was identified in the present study multivariate model as a significant independent risk factor of falling among old age persons. This would undoubtedly be due to that this score is the summative outcome of poor physical health. In congruence with this, several systematic reviews with meta-analyses provided a strong evidence of the positive effect of exercise training interventions in decreasing the risk of falling among old age people (21–23). Moreover, exercise interventions associated with lifestyle changes proved to have a high effectiveness in reducing the risk of falling in the elderly (24).

Concerning the health problems related to the occurrence of fall incidents among old age persons, the current study bivariate analyses demonstrated significant associations with their affection by certain chronic ailments such as ischemic heart disease, hypertension, dyslipidemia, stroke, and diabetes mellitus, as well as osteoporosis, cataract, arthritis, and history of fracture hip. These chronic conditions certainly have negative impacts on the elderly' physical and mental abilities, thereby increasing their risk of falling. In fact, the number of health problems was identified in the multivariate analyses as an independent significant positive predictor of the risk of falling. The findings are in agreement with a recent study in China that examined the relation between the number of chronic diseases and falling in the elderly population (25).

The multivariate analysis also identified stroke and diabetes mellitus as significant independent predictors of falling among old age persons. The influence of stroke is quite understandable given its negative impacts on the affected person's physical as well as mental abilities. In congruence with this, a study in Japan identified stroke as an independent predictor of the risk of falling among elderly (26). As for diabetes mellitus, it might increase the risk of falling due to associated neuropathy that may make the diabetic person more vulnerable to accidents and injuries. Moreover, diabetic patients may have impaired reactive balance to disequilibrium as demonstrated in a study in the United States, thus increasing their risk of falling (27).

The present study bivariate analyses also showed that the old age persons in the fall group reported significantly higher rates of almost all symptoms related to various chronic diseases. This again reflects their poor health status. However, the only symptom confirmed in the multivariate analysis was that of dizziness. This is quite conceivable given that it can lead to disequilibrium and the old age person is often unable to rapidly regain his/her equilibrium and his/her physical fitness and abilities cannot help in preventing falling. Similar significant relations between dizziness and the risk of falling among elderly were reported in studies in Sweden (28) and Greece (29).

The fear of fall (FOF) was also identified as a major factor affecting the occurrence of falls among the old age persons in the present study. Its role was evident both in bivariate and multivariate analyses. It may act through limiting the old person's physical activities, thus leading to low physical fitness and weak muscles, and may lead to frailty and even sarcopenia. In line with this, a study of elderly in Iran clarified that the fear of fall among them is associated with disturbance of their activities of daily living, with consequent negative impact on their quality of life (30). Furthermore, Qin et al. (31) in a study in China demonstrated a significant association between FOF and frailty among old age people.

The current study results revealed that the old age persons who experienced falling had higher rates of medication use compared with the non-fallers in the bivariate analyses. They also had higher rates of administration of polypharmacy. The influence of polypharmacy as a risk factor predicting falls in the old age people has been previously documented in the literature (32, 33). Yet, the effect of polypharmacy on the occurrence of fall was not confirmed in the current study multivariate analysis. This might be explained by that the effect of the disease itself as stroke or diabetes mellitus was more important than that of related medications.

The present study multivariate regression analysis produced a model that depends on the history of stroke and diabetes mellitus, the fear of fall and dizziness, the total number of health problems and the score of difficulty in performing physical activities. A similar model was developed by Gade et al. (28) in Denmark. The risk factors included in their model were old age person's level of education, feeling dizzy, alcohol intake, history of previous falls, perceived risk of falling, disabilities, and symptoms of depression. The differences with the present study model might be attributed to the differences in the study designs, where the Denmark study was a cohort one, in addition to the socio-demographic differences between the two studies such as the level of education and alcohol consumption. However, as highlighted by Seaman et al. (34), the development of predictive fall models for the elderly is still in “its infancy.” Thus, more research is needed.

Our study has some limitations that could jeopardize its generalizability to the whole Egyptian population. Firstly, the inclusion of participants in the fifth decade of age could make the study results not comparable to many old age studies. Secondly, the convenience sampling could have led to bias in the level of education, which might not reflect the true population figures. Lastly, the sample size was calculated using a relatively large margin of error, which might have led to a small sample size for a prevalence study; however, the sample size was large enough for the multivariate analysis.

## Conclusion and recommendations

The prevalence of falls among old age persons in the studied community is not alarmingly high, yet it could be reduced since the target should be “no-falls.” The risk is highest among stroke patients. The study recommends rehabilitation & community interventions to train and educate old age people, especially those at risk such as stroke and diabetic patients, and those with dizziness to improve their physical fitness and reduce the fear of fall among them. The developed prediction model needs to be validated through longitudinal studies.

## Data availability statement

The original contributions presented in the study are included in the article/supplementary material, further inquiries can be directed to the corresponding author.

## Ethics statement

The studies involving human participants were reviewed and approved by IRB of the American University in Cairo (IRB-AUC). The patients/participants provided their written informed consent to participate in this study.

## Author contributions

AE, MS, OM, and AA: drafted the first version, conceptualized the idea, conducted data collection, and analysis. MS: funding, conceptualization, and final revision. All authors contributed to the article and approved the submitted version.
